# Fat Mass Index (FMI) as a Trustworthy Overweight and Obesity Marker in Mexican Pediatric Population

**DOI:** 10.3390/children7030019

**Published:** 2020-03-10

**Authors:** Melchor Alpízar, Tamara Daniela Frydman, José de Jesús Reséndiz-Rojas, Miguel Alejandro Trejo-Rangel, Jesús Manuel De Aldecoa-Castillo

**Affiliations:** Specialized Centre for Diabetes, Obesity and Prevention of Cardiovascular Diseases, Mexico City 11650, Mexico; tfrydman@cedopec.com (T.D.F.); jresendez@cedopec.com (J.d.J.R.-R.); alex.pharmath@gmail.com (M.A.T.-R.)

**Keywords:** pediatric obesity, body mass index, adipose tissue

## Abstract

Predictive formulas to estimate body composition in children have been explored for some time, to this date, the most accurate obesity diagnostic tool is to determine fat mass. The aim of this study is to establish cutoff points that allow for a precise interpretation of nutritional status using the Fat Mass Index (FMI) in a Mexican pediatric population. A literature review using PubMed and Cochrane databases was made, searching for research articles on childhood obesity that compared BMI, FM percentage, and FMI, as well as those proposing diagnostic cutoff points. Mathematic formulas and linear regressions were then used to make a proposal on accurate cutoff points for this population. Our findings show that FM percentage is less precise than BMI and FMI in diagnosing obesity, and FMI seems to be a more complete tool for assessment as it differentiates fat and muscle mass of the total body weight. Both BMI and FMI should be considered when clinical evaluations regarding weight, with BMI complementing FMI by establishing fat-free mass. Our proposed cutoff points need to be validated in this population.

## 1. Introduction

**Figure 1:**
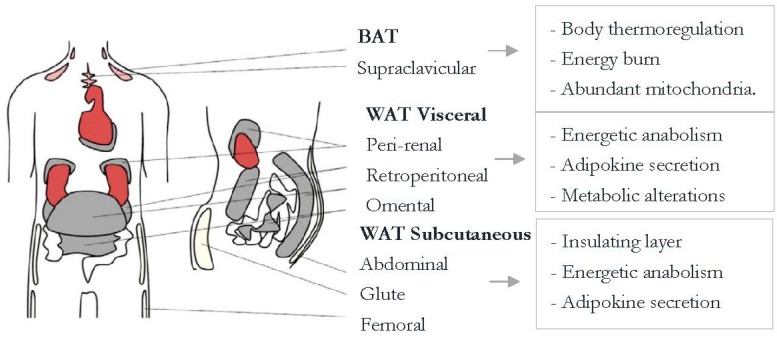
White and brown adipose tissue general information. Brown adipose tissue (BAT) contributes to heat production. BAT adipocytes have several mitochondria and high UCP-1 (Uncoupling Protein-1) expression, which relates to thermogenesis. Therefore, this type of tissue is thought of as a reducer of obesity. White adipose tissue (WAT), on the other hand, saves energy and secretes adipokines to regulate energy homeostasis. (Choe et al. 2016) [2].

Fat mass (FM) distribution and adipose tissue dysfunction are the most efficient predictors of insulin resistance (IR) and related complications, even more so than Body Mass Index (BMI). Adipose tissue dysfunction can be determined through local inflammation and lipid metabolism alterations [1]. To explain the characteristics of obesity, we first need to know that adipose tissue is an endocrine organ that is central in energetic homeostasis regulation. There are two types of adipose tissue (Figure 1), white (WAT)—known for its lipid anabolism- and brown (BAT)—known for its thermogenic effect [2].

Chronic overnutrition elicits an uncontrolled inflammatory response that leads to metabolic complications such as IR [2]. This chronic illness targets the vulnerable pediatric population worldwide. Children not only develop adipose hypertrophy but hyperplasia as well, and they maintain this increased number of adipose cells throughout their lives, meaning they will have an easier time holding on to lipids until puberty when this hyperplasia decreases [3]. These changes affect adipokine secretion, apoptosis, and local hypoxia.

Obesity is defined in general terms as a chronic illness characterized by an excess of body fat that arises mainly from caloric energy imbalance [3].

Cells that make up the stromal vascular fraction are involved in an adaptive process that becomes dysregulated by obesity [2]. Visceral obesity is more dangerous than subcutaneous because it has more inflammatory activity (IL-6), which in turn switches lipid storage on [1]. The permanent inflammatory state comes from chronic adipocyte dysfunction is characterized by three main features: site (visceral fat accumulation that leads to organ damage), size (hypertrophic adipocytes that absorb more fat), and cyte (referring to the cytokines and immunological cells such as macrophages that respond to and prolong the inflammation) [3]. Between 25% to 30% of obese patients are metabolically healthy obese individuals (who have more subcutaneous than visceral fat), but they aren’t exempt from developing cardio metabolic illness and even though they might not have metabolic syndrome (MetS), their health risk is still greater than those of normal weight.

There is still much controversy about which indicator is ideal for measuring overweight or obesity. Some authors are inclined towards the use of FM%, asserting that it has a high correlation with BMI [4], however, as height is not taken into account, the use of FM % is debated. Recent studies refer to the current need for the accurate assessment of body composition for a complete follow up in weight gain and weight loss scenarios. Total body weight is no longer reliable on its own for a full assessment [5].

This paper aims to review whether Fat Mass Index (FMI) is a reliable indicator of overweight and obese patients, especially during childhood. This objective comes mainly from the fact that BMI, the standard measure of these conditions, can sometimes miss cases where body composition is unhealthy even though the index classifies the patient in a normal weight category.

### Childhood Obesity in the Mexican Population

During childhood, obesity is a serious risk factor for the development of several comorbidities in psychological, cardiovascular, metabolic, and orthopedic areas [6]. The National Survey of Nutrition and Health (ENSANUT) conducts a census every few years to determine the health and nutritional status of the Mexican population. The results of 2016 show that children between 5 and 11 years old have a 33.2% prevalence of obesity, a percentage that remains unchanged since 2012 [7]. This may be due in part to the lack of general standards that could determine accepted cutoff points for weight, height, and anthropometric measures associated with a healthy child with optimal dietary habits. Current proposals are based on studies made in 1975 (Dr. Ramos Galvan), 1977 (National Center for Health Statistics—NCHS) and 2000 (National Health and Nutrition Examination Survey—NHANES), however, these studies do not base their optimal percentiles in a model health depiction [8].

The Center for Disease Control and Prevention (CDC) has the most complete growth and development curves that include weight for age, height for age, and BMI for age as birth and up to 20 years of age. Even so, these curves are based on the previously mentioned NCHS and NHANES studies, where only the USA population is considered (a population with the highest obesity numbers worldwide during those years).

**Figure 2:**
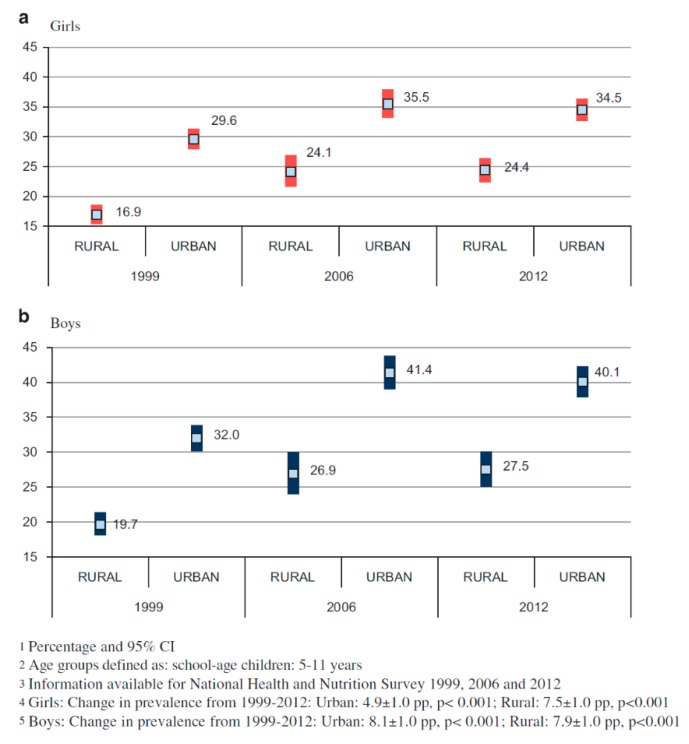
Overweight and obesity prevalence in school aged Mexican girls and boys. [9].

There are not a lot of studies in this regard, however, Hernández-Cordero et al. (2017) published a combined prevalence graph of overweight and obesity in school-age girls and boys by area of residence and year (Figure 2). This showed a significant rise in BMI for this population between 1988 and 2012.

These results show that Mexican children have among the highest prevalence in these categories when compared to similar populations such as Cuba (17.3% in 2003) and Colombia (25.2% in 2005) [9].

As for BMI cutoff points to accurately diagnose overweight and obesity in this population, a study by Mendoza Pablo et al. in 2015 compared references from the WHO, CDC, and IOTF (International Obesity Task Force) and found sensibilities of 57.6, 53.5, and 40.4 respectively for each reference. Specificity ranked higher with percentages between 91.6 and 97.5. However, more studies like this one are needed to determine a definite system to diagnose weight problems in Mexican children [10].

The most recent publication in this regard, made in 2007 by the World Health Organization, includes growth curves for worldwide use, with BMI for age as the indicator of nutritional status.

**Figure 3:**
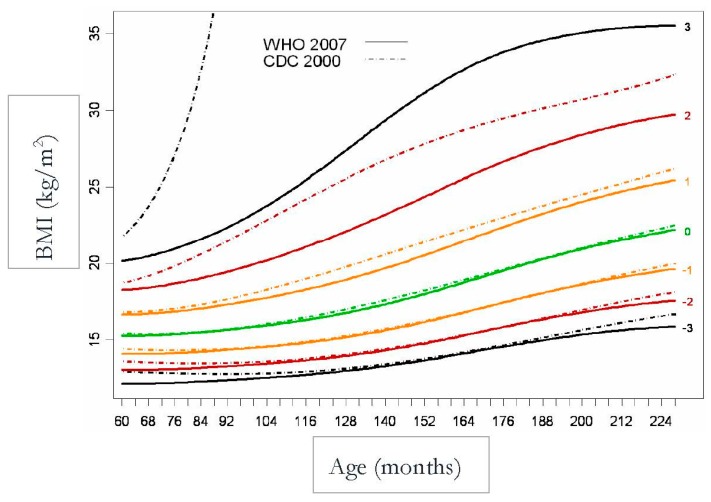
WHO and CDC Comparison (Boys).

**Figure 4:**
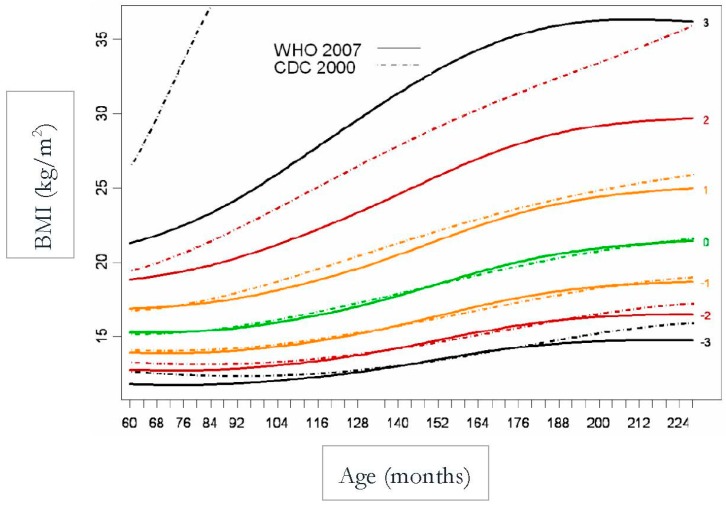
WHO and CDC Comparison (Girls).

In Figure 3 and Figure 4, there is a huge difference between WHO and CDC values that is mainly due to the inclusion criteria (de Onis et al. 2007) [8].

**Figure 5:**
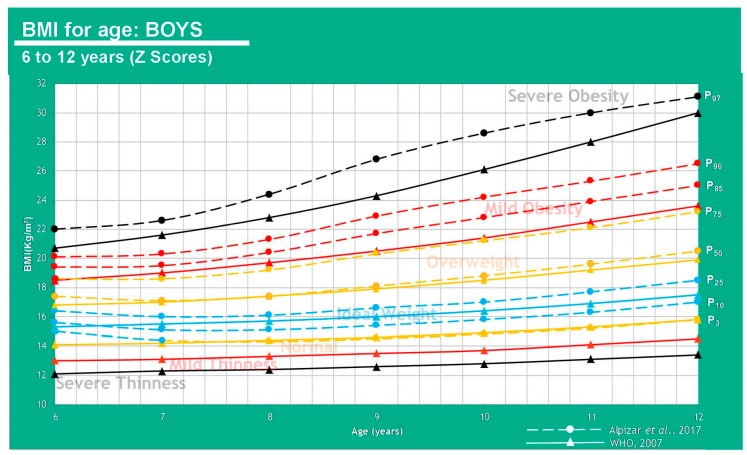
Comparison between average weight in the Mexican population (Alpízar, et al.) and the WHO reference in boys of 6–12 years of age.

**Figure 6:**
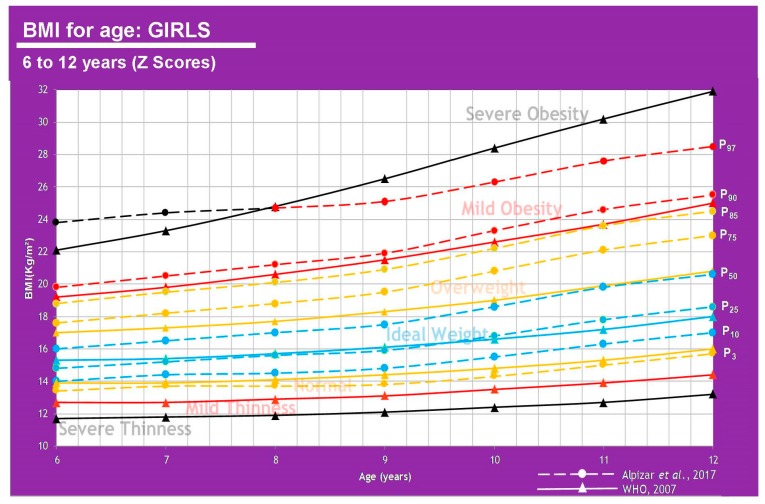
Comparison between average weight in the Mexican population (Alpízar, et al) [7] and the WHO reference in girls of 6–12 years of age.

Mexican population has not yet set a standard to determine the ideal BMI for age in children, however, a study conducted by Alpizar et al. in 2017 set out to measure 3514 healthy subjects to define percentiles for this population. This paper published body composition curves for this same population, evaluating 2026 boys and 1488 girls aged 6–12 years old. This study measured body weight and height to calculate BMI, and total body fat percentage derived from skinfold thickness to help determine fat mass index and fat-free mass index. Skinfold thickness was measured three times in the triceps and subscapular area on the left side always with a caliper Lange model C-130. This study was one of the first efforts to create smoothed body composition percentile curves in this age group, and it also helped strengthen the value of fat mass index over total body weight by correcting by height [7]. That being said, these results are not the ideal standard, nor was that the goal unlike in the WHO study, but instead to capture an actual representation of the population measured. The comparison is shown in Table 1 as well as Figure 5 and Figure 6. 

However, results are different when we base our comparison on FM percentage (25% for boys, 30% for girls) vs. BMI [4,7].

**Figure 7:**
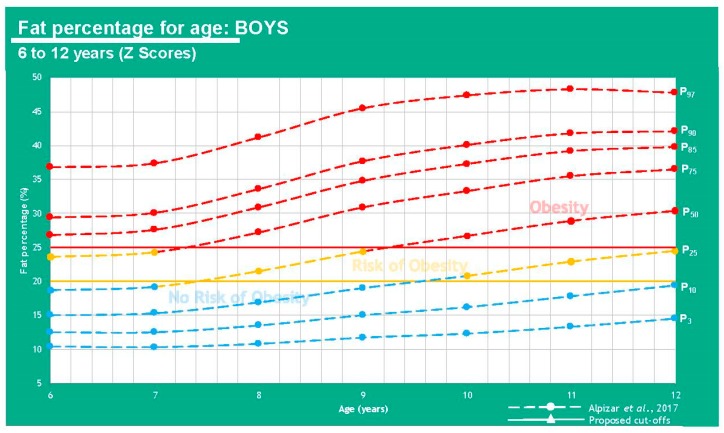
Comparison between results from Alpízar, et al. [7] and the proposed cutoffs for obesity using fat percentage in boys of 6–12 years of age.

**Figure 8:**
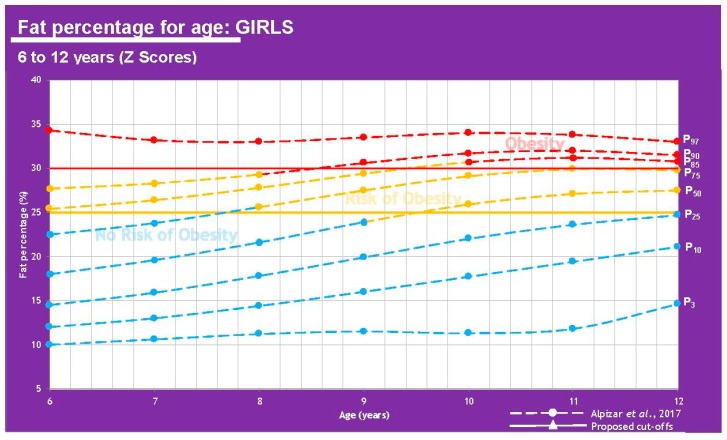
Comparison between results from Alpízar, et al. [7] and the proposed cutoffs for obesity using fat percentage in girls of 6–12 years of age.

The information displayed in Table 2 shows that FM% values give a different diagnosis than BMI, as the latter can underestimate some cases where children are overweight or obese. It can be said that an FM higher than 20% in boys and 25% in girls represents a cardiometabolic risk which is why these cutoff points can be used to define the overweight class. There is a clear association between weight status and anthropometric parameters. These measurements have been used for several years now to determine a nutritional picture and the more specific they get, the more insight physicians have towards body composition and treatment plans in this regard [11]. A graphic representation can be viewed in Figure 7 and Figure 8. 

## 2. Materials and Methods

A systematic review of online literature was made. The search criteria used were from scientific papers focused on Mexican pediatric population, with significant results involving Body Mass Index, and adipose tissue determinants for overweight and obesity diagnosis. Keywords used include fat mass, cutoffs, Mexican, pediatric population, body mass index, and fat mass percentage. Longitudinal studies were preferred. Databases used were PubMed and Cochrane. No studies were eliminated based on their language or nationality. The type of population selected was restricted to under the age of 20. With the information gathered and synthesized, the proposal of cutoff points for a more effective diagnosis in this population was made, even though it has not been validated. This article complies with the International Committee of Biomedical Journal Editors, the General Assembly of the World Medical Association and the World Medical Association Declaration of Helsinki.

### 2.1. BMI or FM Percentage as Diagnostic Tools for Overweight and Obesity

The latest classification published by the WHO diagnoses overweight with a BMI of 25–29.9 kg/m^2^, class 1 obesity with a BMI of 30–34.9 kg/m^2^, class 2 obesity with a BMI of 35–39.9 kg/m^2^ and class 3 with a BMI of 40 kg/m^2^ or higher [12]. This measure is the most used as well as the most controversial due to its poor predictive capacity to determine fat mass, taking into consideration total weight and height as the only variables and leaving aside body composition as a part of the criteria [13].

This leads to a definition of a different diagnosis called normal weight obesity, which is characterized as a normal BMI or bodyweight with a high fat mass percentage that leads to a metabolic dysfunction like the one caused by obesity as well as the high predisposition for cardiovascular disease [14]. This is especially true for some cultures more than others as recent evidence suggests that Asian and Mexican populations tend to a higher fat mass percentage than Europeans do, which may be due to epigenetic variations as well as lifestyle habits [4,12].

A study completed in 2009 studying cardiac insufficiency prognostic factors found that, while high BMI and muscular mass were associated with low N-terminal pro-B-type natriuretic peptide, high-fat mass percentage showed the association to higher RCP and a lower physical capability. This led to the conclusion that BMI in this population was directly in sync with muscle mass and not with fat mass, and therefore not a very good identifier for obesity [15].

Madeira et al. 2013, reviewed several articles discussing the close relationship between normal weight obesity and cardiometabolic risk, low HDL, high waist circumference, hyperglycemia, and hypertriglyceridemia. They also evaluated 1222 subjects of 23–25 years of age and found that normal weight obesity (defined as a normal BMI with at least 25% body fat for men and 30% for women) was highly associated with insulin resistance and metabolic syndrome development [14].

Pediatric population still does not have standard cutoff points to determine excess body fat, however, studies have reported the same 25% and 30% for boys and girls respectively to be associated with risk factors such as high cholesterol, blood pressure, and triglycerides as well as low HDL and cardiovascular disease [4,7].

### 2.2. Fat Mass Index as a Diagnostic Tool

The concept of FMI compared to BMI, considering a bicompartmental model, seems to be a more accurate tool in overweight/excess fat.

The equation to determine FMI is the following:

$$\mathrm{FMI}\;=\;\frac{\mathrm{FM}\;(\mathrm{kg})}{\mathrm{height}\;(m^{2})}$$

FMI: fat mass index; FM: fat mass.

To determine FM in net kilograms, the next equation is needed:

$$\mathrm{FM}\;(\mathrm{kg})\;=\;\frac{\left[\mathrm{FM}\%\;\times \;\mathrm{BW}\;(\mathrm{kg})\right]}{100}$$

FM%: fat mass percentage; BW: body weight.

The scarce use of this index is probably due to a lack of standard cutoff points to determine deficit or excess fat mass or muscle mass in each patient [16]. However, researchers mostly agree on the high level of precision that the FMI must diagnose adipose hypertrophy with. High FMI has been correlated in teens with hypertriglyceridemia, high risk of cardiac disease, and elevated waist circumference [17].

### 2.3. Calculating Cutoff Points Proposed for Mexican Pediatric Population

During pre-puberty (Tanner stage 1) girls have a higher fat mass percentage than boys (1%–3% variation) and boys have about 0.5 kg of fat-free mass (FFM). At about 10 years old, girls have an average of 2 kg more fat mass and boys have about 1 kg more of muscle mass. Throughout puberty (Tanner stages 2–5) girls will store between 4–6 kg more FM than boys [18,19]. This is important to consider as these changes will affect fat mass percentage and therefore index.

To know the value of FMI ∝ to a BMI class, ideal or normal average fat-free mass index (FFMI) is necessary. Pediatric BMI has been determined by the WHO, so the question is how to calculate FFMI analogous to ideal/normal weight BMI. A reference used for this purpose is described below in Table 3. 

The risk of bias was determined with the RTI Item Bank on Risk of Bias and Precision of Observational Studies [21].

We focused on the values proposed by Freedman et al. 2005 (Table 3) as they cover ages 6–18. With the data reported, several linear regressions were carried out considering mean age as the independent variable and FFMI (low, average, and high) as the dependent variables. Three regressions were performed for girls and 3 for boys. The goal was to be able to determine FFMI using the regression analysis at any age and therefore avoid age groups. Given that BMI is the sum of FFMI and FMI, to determine FMI at any BMI stage we proceeded to subtract the calculated FFMI from the BMI at a given stage (ideal FFMI from ideal BMI and so on).

To determine FMI ∝ to any BMI class—considering that BMI is the sum of FFMI and FMI—we came up with the formulas:

$$x=BMI\;\left(ideal\right)-{FFMI}_{i}\;$$

where x: FMI ∝ to ideal BMI (kg/m^2^); BMI: Body Mass Index (kg/m^2^); FFMI_i_: Ideal Fat Free Mass Index (kg/m^2^).

$$y=BMI\;\left(classes\;beneath\;ideal\right)-{FFMI}_{bi}$$

where y: FMI ∝ to any BMI class beneath ideal (kg/m^2^); BMI: Body Mass Index (kg/m^2^); FFMI_bi_: beneath ideal Fat Free Mass Index (kg/m^2^).

$$z=BMI\;\left(classes\;above\;ideal\right)-{FFMI}_{ai}$$

where z: FMI ∝ to any BMI class above ideal (kg/m^2^); BMI: Body Mass Index (kg/m^2^); FFMI_ai_: above ideal Fat Free Mass Index (kg/m^2^).

**Figure 9:**
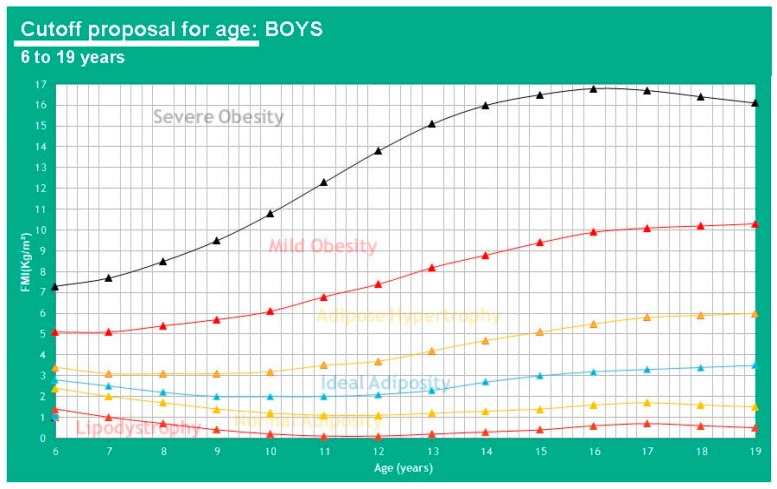
Proposed cutoffs for FMI in boys of 6-19 years of age.

**Figure 10:**
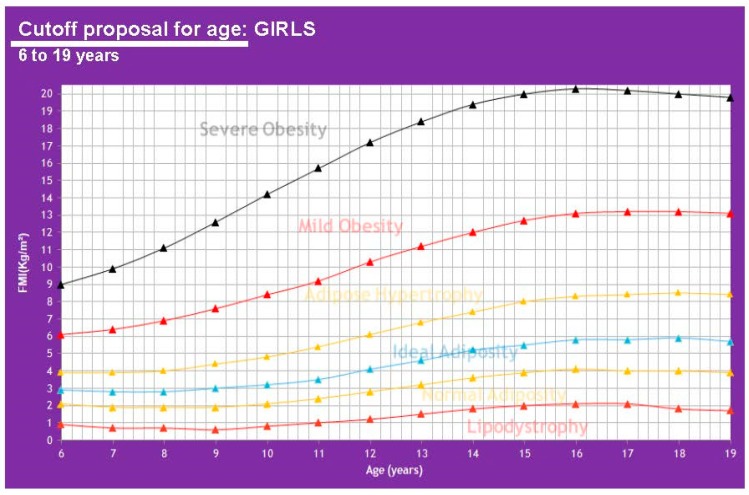
Proposed cutoffs for FMI in girls of 6-19 years of age.

This allows us to know fat mass by subtracting FFMI from BMI. The references shown on Table 4 were used for the adult analysis. Table 5 shows FMI values ∝ to any BMI class in the pediatric population and Table A2 (Appendix A) as well for adults. To begin calculating cutoff points for adults, we start from the BMI reference of an ideal adult index of 22 kg/m^2^ [22]. The proposed cutoffs for FMI by age in a pediatric population can be seen in Figure 9 and Figure 10. 

Finally, for a more accurate diagnosis, FFMI can be deduced from the formula:

$$\mathrm{FFMI}\;(\mathrm{kg}/m^{2})\;=\;BMI-FMI$$

where BMI: Body Mass Index (kg/m^2^), FMI: Fat Mass Index (kg/m^2^).

## 3. Results

When speaking of fat mass cutoff points instead of total body weight, we suggest using lipodystrophy instead of thinness, normal adiposity instead of normal weight, ideal adiposity instead of ideal weight and adipose hypertrophy instead of overweight. It is also important to consider that if girls are at a different Tanner stage for their age, a 0.5% error range in fat mass is allowed.

## 4. Discussion

**Figure 11:**
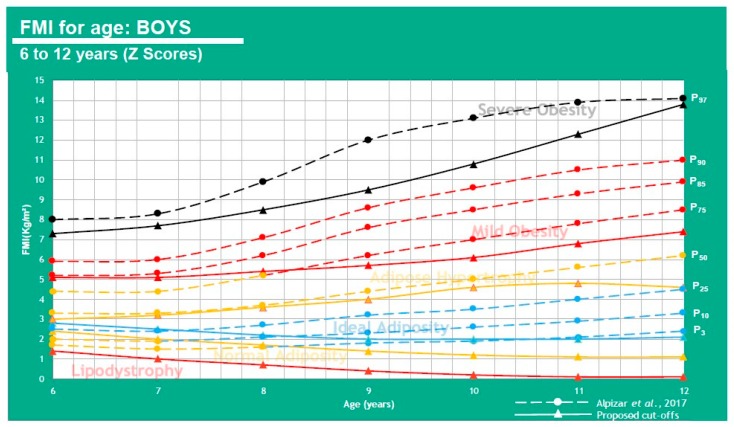
FMI for age in boys of 6–12 years of age.

**Figure 12:**
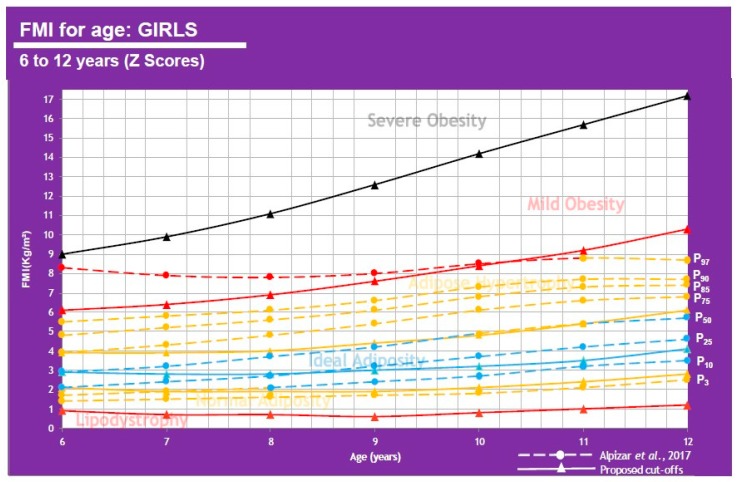
FMI for age in girls of 6–12 years of age.

These cutoff points in Table 6 allow for a comparison of results from Alpizar, et al. 2017 regarding FMI for the Mexican pediatric population and FMI ∝ to BMI class (WHO). A graphic representation can be analyzed using Figure 11 and Figure 12. 

This shows that when using FMI, the boys P50 falls under adipose hypertrophy at all ages except 6 y-o (ideal adiposity) and the girls show ideal adiposity for 6–9 and 12 y-o, but adipose hypertrophy for 10–11 y-o. 25% of boys have adipose hypertrophy at ages 6–8 and mild obesity at ages 9–12. 25% of girls also have adipose hypertrophy at all ages.

In Table 7 and Table A1 (Appendix A), we show BMI, FM%, and FMI values correlated with diagnostic parameters for every class to understand differences between diagnostic tools. It is easy to identify that the P50 of the Mexican populations varies highly when using FM% but remains similar to BMI and FMI although this last one provides a more complete picture by considering fat and muscle mass and not just whole-body weight.

FM and FFM should always be studied when diagnosing weight alterations. Altered FM/FFM can have high-risk consequences including insulin resistance and/or metabolic syndrome due to changes in muscle quantity that lower insulin receptors and alter metabolic homeostasis [25,26]. Being aware of changes to body composition can help physicians to practice preventive medicine and nutrition therapy and have a full diagnosis. This does not mean that BMI is useless, this tool is also helpful as a quick indicator but can sometimes fall short. Several studies have determined FMI ideal cutoff points for diverse populations, Table 8 shows some of their conclusions.

All these results point to a main issue that is that Mexican pediatric populations (as said previously by Alpizar et al. 2017) [7] have a high-fat mass component in body composition and this cannot be overlooked by only analyzing total weight because it means that most of these children will likely have obesity as an adult or otherwise have normal weight obesity and either way they will be at high risk for developing cardiometabolic diseases. Other authors have also arrived at this conclusion, basing their investigations on the published Hattori charts that compare Fat Mass Index and Lean Mass Index with the associated BMI values for every case. These charts show how two subjects with similar BMI can have very different adiposity values and two subjects with similar fat percentages can have a very different BMI [28,29].

Recent results published by the 2018 National Health and Nutrition Survey (ENSANUT) show that 35.6% of the Mexican population between the ages of 5 and 11 are overweight or obese (18.1% and 17.5%, respectively) with practically no change as the 2006 (34.8%) and 2012 (34.4%) surveys [30]. These results are alarming and call for desperate measures in diagnosis improvement for more efficient prevention and treatment.

The FFMI indicator has demonstrated high specificity of 95.8% to 97.6% for the diagnosis of MetS risk [27] so its prompt validation is suggested as a diagnostic tool in the Mexican pediatric population.

## 5. Conclusions

FM percentage turned out to be the least efficient diagnostic indicator to determine overweight and obesity, which makes sense as it does not consider height. BMI and FMI resulted in very similar diagnosis, however, FMI looks like a more trustworthy index because of its consideration of body composition. The precise insight that FMI gives on FM and FFM can guide treatment during weight loss in a way that BMI cannot.

The proposed cutoff points in this review should be validated in this population and compared to health status to determine official classes and improve overweight and obesity diagnosis.

## Figures and Tables

**Table 1:** BMI for age for boys and girls.

Z Scores (6 to 12 Years Old)														
	Z Scores WHO (BMI kg/m^2^)													
Age (Years)	−3 DE (Severe Thinness)		−2 DE (Mild Thinness)		−1 DE (Normal Weight)		Average (Ideal Weight)		+1 DE (Overweight)		+2 DE (Mild Obesity)		+3 DE (Severe Obesity)	
**Boys**	**WHO**	**Alpízar**	**WHO**	**Alpízar**	**WHO**	**Alpízar**	**WHO**	**Alpízar**	**WHO**	**Alpízar**	**WHO**	**Alpízar**	**WHO**	**Alpízar**
6	12.1	-	13.0	-	14.1	-	15.3	P_3,_ P_10_, P_25_	16.8	P_50_	18.5	P_75_, P_85_, P_90_	20.7	P_97_
7	12.3	-	13.1	-	14.2	-	15.5	P_3,_ P_10_, P_25_	17.0	P_50_, P_75_	19.0	P_85_, P_90_	21.6	P_97_
8	12.4	-	13.3	-	14.4	P_3_	15.7	P_10_, P_25_	17.4	P_50_, P_75_	19.7	P_85_, P_90_	22.8	P_97_
9	12.6	-	13.5	-	14.6	P_3_	16.0	P_10_, P_25_	17.9	P_50_, P_75_	20.5	P_85_, P_90_	24.3	P_97_
10	12.8	-	13.7	-	14.9	P_3_	16.4	P_10_, P_25_	18.5	P_50_, P_75_	21.4	P_85_, P_90_	26.1	P_97_
11	13.1	-	14.1	-	15.3	P_3_	16.9	P_10_, P_25_	19.2	P_50_, P_75_	22.5	P_85_, P_90_	28.0	P_97_
12	13.4	-	14.5	-	15.8	P_3_	17.5	P_10_, P_25_	19.9	P_50_, P_75_	23.6	P_85_, P_90_	30.0	P_97_
Girls														
6	11.7	-	12.7	-	13.9	P_3_	15.3	P_10_, P_25_, **P_50_**	17.0	P_75_, P_85_	19.2	P_90_	22.1	P_97_
7	11.8	-	12.7	-	13.9	P_3_	15.4	P_10_, P_25_, **P_50_**	17.3	P_75_, P_85_	19.8	P_90_	23.3	P_97_
8	11.9	-	12.9	-	14.1	P_3_	15.7	P_10_, P_25_, **P_50_**	17.7	P_75_, P_85_	20.6	P_90_, P_97_	24.8	-
9	12.1	-	13.1	-	14.4	P_3_	16.1	P_10_, P_25_, **P_50_**	18.3	P_75_, P_85_	21.5	P_90_, P_97_	26.5	-
10	12.4	-	13.5	-	14.8	P_3_	16.6	P_10_, P_25_, **P_50_**	19.0	P_75_, P_85_	22.6	P_90_, P_97_	28.4	-
11	12.7	-	13.9	-	15.3	P_3_	17.2	P_10_, P_25_, **P_50_**	19.9	P_75_, P_85_	23.7	P_90_, P_97_	30.2	-
12	13.2	-	14.4	-	16.0	P_3_	18.0	P_10_, P_25_, **P_50_**	20.8	P_75_, P_85_	25.0	P_90_, P_97_	31.9	-

Table 1. Comparison between Alpizar et al. (2017) [7] and WHO studies. Average weight in the Mexican population coincides with a Z score of overweight children (WHO) for boys, whereas the girl´s average weight is in the Z score for normal weight (WHO).

**Table 2:** Fat Mass (FM)% in the Mexican pediatric population.

	%FM Based Cutoff Points for Childhood Obesity					
Age (Years)	Percentiles	FM%	Percentiles	FM%	Percentiles	FM%
**Boys**	**No Obesity Risk (<20.0%)**		**Risk of Obesity (20.0–25.0%)**		**Obesity (>25.0%)**	
6	P_3_, P_10_, P_25_, P_50_	10.4–18.7	P_75_	23.6	P_85_, P_90_, P_97_	26.8–36.8
7	P_3_, P_10_, P_25_, P_50_	10.3–19.2	P_75_	24.3	P_85_, P_90_, P_97_	27.6–37.4
8	P_3_, P_10_, P_25_	10.8–16.9	P_50_	21.5	P_75_, P_85_, P_90_, P_97_	27.2–41.2
9	P_3_, P_10_, P_25_	11.7–19.0	P_50_	24.4	P_75_, P_85_, P_90_, P_97_	30.9–45.5
10	P_3_, P_10_	12.3–16.2	P_25_	20.8	P_50_, P_75_, P_85_, P_90_, P_97_	26.7–47.4
11	P_3_, P_10_	13.3–17.8	P_25_	22.9	P_50_, P_75_, P_85_, P_90_, P_97_	28.9–48.3
12	P_3_, P_10_	14.5–19.4	P_25_	24.5	P_50_, P_75_, P_85_, P_90_, P_97_	30.4–47.8
**Girls**	**No Obesity Risk (<25.0 %)**		**Risk of Obesity (25.0–30.0%)**		**Obesity (>30.0%)**	
6	P_3_, P_10_, P_25_, P_50_, P_75_	10.0–22.5	P_85_, P_90_	25.4–27.7	P_97_	34.3
7	P_3_, P_10_, P_25_, P_50_, P_75_	10.6–23.8	P_85_, P_90_	26.4–28.3	P_97_	33.2
8	P_3_, P_10_, P_25_, P_50_	11.2–21.6	P_75_, P_85_, P_90_	25.6–29.3	P_97_	33.0
9	P_3_, P_10_, P_25_, P_50_	11.5–23.9	P_75_, P_85_	27.5–29.4	P_90_, P_97_	30.6–33.5
10	P_3_, P_10_, P_25_	11.3–22.0	P_50_, P_75_	25.9–29.1	P_85_, P_90_. P_97_	30.7–34.0
11	P_3_, P_10_, P_25_	11.8–23.6	P_50_, P_75_	27.1–29.9	P_85_, P_90_. P_97_	31.2–33.8
12	P_3_, P_10_, P_25_	14.6–24.7	P_50_, P_75_	27.5–29.8	P_85_, P_90_. P_97_	30.8–33.0

Table 2. Comparison between Alpizar et al. (2017) [7] vs. cutoff points proposed for obesity. P_50_ for boys ages 6–7 has no risk of obesity, ages 8–9 are at risk, and 10–12 are obese. However, girls of ages 6–9 have no risk and ages 10–12 are at risk.

**Table 3:** FFMI analogous to normal weight BMI (pediatric patients).

Different Population Studies													
Reference	*n* Total	*n* Men	*n* Women	6–8 y-o		9–11 y-o		12–14 y-o		15–18 y-o		Population	Risk of Bias
				Boys	Girls	Boys	Girls	Boys	Girls	Boys	Girls		
Freedman et al. (2005) [20]	1104	578	526	12.5–14.0	12.1–13.2	13.1–14.5	12.3–14.2	14.7–17.0	13.5–15.2	17.4–18.3	14.3–15.8	USA	Low

*n*: population size; FFMI: fat-free mass index analogous to normal weight BMI; N/D: not determined.

**Table 4:** Studies on FFMI analogous to normal weight BMI.

Different Population Studies										
Reference	*n* Total	*n* Men	*n* Women	Ages (Years)	Population	FFMI (kg/m^2^) Men, Ranges	FFMI (kg/m^2^) Women, Ranges	FFMI (kg/m^2^) Men, Mean	FFMI (kg/m^2^) Women, Mean	Risk of Bias
Kudsk et al. (2017) [23]	16,000	8000	8000	12–90	USA	N/D	N/D	19.1	15.9	Moderate
Jensen et al. (2019) [24]	3072	1554	1518	18–87	Germany, Japan and Mexico	16.8–19.0	14.1–15.9	17.9	15.0	Low

*n*: population size; FFMI: Fat Free Mass Index analogous to normal weight BMI; N/D: not determined.

**Table 5:** Fat Mass Index (FMI) classification proposal for the pediatric Mexican population.

	FMI (kg/m^2^) ∝ to BMI (kg/m^2^) Proposal											
Age (Years)/Tanner Stage	−2 DE (Thinness–Lipodystrophy)		−1 DE (Normal Weight–Normal Adiposity)		Mean (Ideal Weight–Ideal Adiposity)		+1 DE (Overweight–Adipose Hypertrophy)		+2 DE (Mild Obesity)		+3 DE (Severe Obesity)	
**Girls**	**BMI**	**FMI**	**BMI**	**FMI**	**BMI**	**FMI**	**BMI**	**FMI**	**BMI**	**FMI**	**BMI**	**FMI**
6 y-o												
I-II	12.7	0.9	13.9	2.1	15.3	2.9	17.0	3.9	19.2	6.1	22.1	9.0
7 y-o												
I-II	12.7	0.7	13.9	1.9	15.4	2.8	17.3	3.9	19.8	6.4	23.3	9.9
8 y-o												
II-III	12.9	0.7	14.1	1.9	15.7	2.8	17.7	4.0	20.6	6.9	24.8	11.1
9 y-o												
II-III	13.1	0.6	14.4	1.9	16.1	3.0	18.3	4.4	21.5	7.6	26.5	12.6
10 y-o												
II-III	13.5	0.8	14.8	2.1	16.6	3.2	19.0	4.8	22.6	8.4	28.4	14.2
11 y-o												
II-III	13.9	1.0	15.3	2.4	17.2	3.5	19.9	5.4	23.7	9.2	30.2	15.7
12 y-o												
III-IV	14.4	1.2	16.0	2.8	18.0	4.1	20.8	6.1	25.0	10.3	31.9	17.2
13 y-o												
III-IV	14.9	1.5	16.6	3.2	18.8	4.6	21.8	6.8	26.2	11.2	33.4	18.4
14 y-o												
III-V	15.4	1.8	17.2	3.6	19.6	5.2	22.7	7.4	27.3	12.0	34.7	19.4
15 y-o												
IV-V	15.9	2.0	17.8	3.9	20.2	5.5	23.5	8.0	28.2	12.7	35.5	20.0
16 y-o												
IV-V	16.2	2.1	18.2	4.1	20.7	5.8	24.1	8.3	28.9	13.1	36.1	20.3
17 y-o												
IV-V	16.4	2.1	18.4	4.1	21.0	5.8	24.5	8.4	29.3	13.2	36.3	20.2
18 y-o												
V	16.4	1.8	18.6	4.0	21.3	5.9	24.8	8.5	29.5	13.2	36.3	20.0
19 y-o												
V	16.5	1.7	18.7	3.9	21.4	5.7	25.0	8.4	29.7	13.1	36.4	19.8
Boys												
6 y-o	13.0	1.4	14.1	2.4	15.3	2.8	16.8	3.4	18.5	5.1	20.7	7.3
7 y-o	13.1	1.0	14.2	2.0	15.5	2.5	17.0	3.1	19.0	5.1	21.6	7.7
8 y-o	13.3	0.7	14.4	1.7	15.7	2.2	17.4	3.1	19.7	5.4	22.8	8.5
9 y-o	13.5	0.4	14.6	1.4	16.0	2.0	17.9	3.1	20.5	5.7	24.3	9.5
10 y-o	13.7	0.2	14.9	1.2	16.4	2.0	18.5	3.2	21.4	6.1	26.1	10.8
11 y-o	14.1	0.1	15.3	1.1	16.9	2.0	19.2	3.5	22.5	6.8	28.0	12.3
12 y-o	14.5	0.1	15.8	1.1	17.5	2.1	19.9	3.7	23.6	7.4	30.0	13.8
13 y-o	14.9	0.2	16.4	1.2	18.2	2.3	20.8	4.2	24.8	8.2	31.7	15.1
14 y-o	15.5	0.3	17.0	1.3	19.0	2.7	21.8	4.7	25.9	8.8	33.1	16.0
15 y-o	16.0	0.4	17.6	1.4	19.8	3.0	22.7	5.1	27.0	9.4	34.1	16.5
16 y-o	16.5	0.6	18.2	1.6	20.5	3.2	23.5	5.5	27.9	9.9	34.8	16.8
17 y-o	16.9	0.7	18.8	1.7	21.1	3.3	24.3	5.8	28.6	10.1	35.2	16.7
18 y-o	17.3	0.6	19.2	1.6	21.7	3.4	24.9	5.9	29.2	10.2	35.4	16.4
19 y-o	17.6	0.5	19.6	1.5	22.2	3.5	25.4	6.0	29.7	10.3	35.5	16.1

Table 5. Proposed interpretation of values to diagnose FM related stages in 6–19-year-olds. This correlation allows the use of FMI to diagnose considering FM and not just total body weight.

**Table 6:** FMI in a Mexican pediatric population.

	Childhood Obesity Cutoff Points Using FMI (kg/m^2^)											
Age (Years)	−2 DE (Lipodystrophy)		−1 DE (Normal Adiposity)		Mean (Ideal Adiposity)		+1 DE (Adipose Hypertrophy)		+2 DE (Mild Obesity)		+3 DE (Severe Obesity)	
**Boys**	**FMI** **∝**	**Alpízar**	**FMI** **∝**	**Alpízar**	**FMI** **∝**	**Alpízar**	**FMI** **∝**	**Alpízar**	**FMI** **∝**	**Alpízar**	**FMI** **∝**	**Alpízar**
6	1.4	-	2.4	P_3_, P_10_	2.8	P_25_, P_50_	3.4	P_75_	5.1	P_85_, P_90_	7.3	P_97_
7	1.0	-	2.0	P_3_, P_10_	2.5	P_25_	3.1	P_50,_ P_75_	5.1	P_85_, P_90_	7.7	P_97_
8	0.7	-	1.7	P_3_	2.2	P_10_, P_25_	3.1	P_50,_ P_75_	5.4	P_85_, P_90_	8.5	P_97_
9	0.4	-	1.4	-	2.0	P_3_, P_10_	3.1	P_25_, P_50_	5.7	P_75_, P_85_, P_90_	9.5	P_97_
10	0.2	-	1.2	-	2.0	P_3_, P_10_	3.2	P_25_, P_50_	6.1	P_75_, P_85_, P_90_	10.8	P_97_
11	0.1	-	1.1	-	2.0	P_3_, P_10_	3.5	P_25_, P_50_	6.8	P_75_, P_85_, P_90_	12.3	P_97_
12	0.1	-	1.1	-	2.1	P_3_, P_10_	3.7	P_25_, P_50_	7.4	P_75_, P_85_, P_90_	13.8	P_97_
Girls												
6	0.9	-	2.1	P_3_, P_10_, P_25_	2.9	P_50_	3.9	P_75_, P_85_, P_90_	6.1	P_97_	9.0	-
7	0.7	-	1.9	P_3_, P_10_	2.8	P_25_, P_50_	3.9	P_75_, P_85_, P_90_	6.4	P_97_	9.9	-
8	0.7	-	1.9	P_3_	2.8	P_10_, P_25_, P_50_	4.0	P_75_, P_85_, P_90_	6.9	P_97_	11.1	-
9	0.6	-	1.9	P_3_	3.0	P_10_, P_25_, P_50_	4.4	P_75_, P_85_, P_90_	7.6	P_97_	12.6	-
10	0.8	-	2.1	P_3_	3.2	P_10_, P_25_	4.8	P_50,_ P_75_, P_85_, P_90_	8.4	P_97_	14.2	-
11	1.0	-	2.4	P_3_	3.5	P_10_, P_25_	5.4	P_50,_ P_75_, P_85_, P_90,_ P_97_	9.2	-	15.7	-
12	1.2	-	2.8	P_3_	4.1	P_10_, P_25,_ P_50_	6.1	P_75_, P_85_, P_90,_ P_97_	10.3	-	17.2	-

Table 6. Results from Alpizar et al. (2017) [7] and FMI cutoff points proposed as proportional to BMI classes.

**Table 7:** BMI, FM%, and FMI comparison in a Mexican pediatric population (short version).

	BMI (kg/m^2^) Cutoff Points, FM% and FMI (kg/m^2^) vs. Mexican Pediatric Population Values		
Age (Years)	50th Percentile		
**Boys**	**BMI**	**FM%**	**FMI**
6	Overweight	No risk of obesity	Ideal adiposity
7	Overweight	No risk of obesity	Adipose hypertrophy
8	Overweight	At risk	Adipose hypertrophy
9	Overweight	At risk	Adipose hypertrophy
10	Overweight	Obesity	Adipose hypertrophy
11	Overweight	Obesity	Adipose hypertrophy
12	Overweight	Obesity	Adipose hypertrophy
Girls			
6	Ideal weight	No risk of obesity	Ideal adiposity
7	Ideal weight	No risk of obesity	Ideal adiposity
8	Ideal weight	No risk of obesity	Ideal adiposity
9	Ideal weight	No risk of obesity	Ideal adiposity
10	Ideal weight	At risk	Adipose hypertrophy
11	Ideal weight	At risk	Adipose hypertrophy
12	Ideal weight	At risk	Ideal adiposity

Table 7. Comparison between results from Alpizar et al. 2017 [7] and FMI proposed cutoff points proportional to BMI classes.

**Table 8:** FMI studies and their relationship with obesity and cardiometabolic illness.

Different Population Studies										
Reference	*n* Total	*n* Men	*n* Women	Ages (Years)	Population	FMI (kg/m^2^) Men	FMI (kg/m^2^) Women	Notes	Technique Used to Determine Adiposity	Risk of Bias
Van Itallie et al. (1990) [16]	192	192	0	20.59	USA	8.3–9.7	N/D	P_95_, FM excess (obesity).	Electromagnetic scanning instrument (EM-SCAN)	Moderate
Liu et al. (2013) [13]	1698	1105	593	20–79	China	7.0	7.9	MetS risk	Bioelectrical impedance analysis	Moderate
Morais et al. (2016) [17]	403	185	218	10–14	Brazil	4.9–5.3	6.2–8.5	Cardiovascular risk	Bioelectrical impedance analysis	Moderate
Aerobics Center Longitudinal Study, ACLS (2016) [25]	60,335	44,234	16,101	43 (media)	USA	10.4; 11.9	12.0; 12.9	Cardiovascular risk; class II obesity in men and class I in women	Skinfold thicknesses–sum of 7 skinfold measures or hydrostatic weighting	Low
FUPRECOL Study (2017) [27]	1687	617	1070	18–35	Colombia	6.97	11.86	MetS risk	Bioelectrical impedance analysis	Low

*n*: population size; MetS: metabolic syndrome; FMI: Fat Mass Index; FM: fat mass; N/D: not determined; P_95_: 95th percentile.

## Appendix A

**Table A1:** BMI, FM%, and FMI comparison in the Mexican pediatric population (extended).

	BMI (kg/m^2^), FM%, and FMI Cutoff Points vs. Mexican Pediatric Population Values																							
Age (Years)	3rd Percentile			10th Percentile			25th Percentile			50th Percentile			75th Percentile			85th Percentile			90th Percentile			97th Percentile		
**Boys**	**BMI**	**% FM**	**FMI**	**BMI**	**% FM**	**FMI**	**BMI**	**% FM**	**FMI**	**BMI**	**% FM**	**FMI**	**BMI**	**% FM**	**FMI**	**BMI**	**% FM**	**FMI**	**BMI**	**% FM**	**FMI**	**BMI**	**% FM**	**FMI**
6	IW	NR	NA	IW	NR	NA	IW	NR	IA	OW	NR	IA	OL	RO	AH	MO	O	MO	MO	O	MO	SO	O	SO
7	IW	NR	NA	IW	NR	NA	IW	NR	IA	OW	NR	AH	OW	RO	AH	MO	O	MO	MO	O	MO	SO	O	SO
8	NW	NR	NA	IW	NR	IA	IW	NR	IA	OW	RO	AH	OW	O	AH	MO	O	MO	MO	O	MO	SO	O	SO
9	NW	NR	IA	IW	NR	IA	IW	NR	AH	OW	RO	AH	OW	O	MO	MO	O	MO	MO	O	MO	SO	O	SO
10	NW	NR	IA	IW	NR	IA	IW	RO	AH	OW	O	AH	OW	O	MO	MO	O	MO	MO	O	MO	SO	O	SO
11	NW	NR	IA	IW	NR	IA	IW	RO	AH	OW	O	AH	OW	O	MO	MO	O	MO	MO	O	MO	SO	O	SO
12	NW	NR	IA	IW	NR	IA	IW	RO	AH	OW	O	AH	OW	O	MO	MO	O	MO	MO	O	MO	SO	O	SO
Girls																								
6	NW	NR	NA	IW	NR	NA	IW	NR	NA	IW	NR	IA	OW	NR	AH	OW	RO	AH	MO	RO	AH	SO	O	MO
7	NW	NR	NA	IW	NR	NA	IW	NR	IA	IW	NR	IA	OW	NR	AH	OW	RO	AH	MO	RO	AH	SO	O	MO
8	NW	NR	NA	IW	NR	IA	IW	NR	IA	IW	NR	IA	OW	RO	AH	OW	RO	AH	MO	RO	AH	MO	O	MO
9	NW	NR	NA	IW	NR	IA	IW	NR	IA	IW	NR	IA	OW	RO	AH	OW	RO	AH	MO	O	AH	MO	O	MO
10	NW	NR	NA	IW	NR	IA	IW	NR	IA	IW	RO	AH	OW	RO	AH	OW	O	AH	MO	O	AH	MO	O	MO
11	NW	NR	NA	IW	NR	IA	IW	NR	IA	IW	RO	AH	OW	RO	AH	OW	O	AH	MO	O	AH	MO	O	AH
12	NW	NR	NA	IW	NR	IA	IW	NR	IA	IW	RO	IA	OW	RO	AH	OW	O	AH	MO	O	AH	MO	O	AH

Table A1. Alpizar et al. 2017 [7] results compared to FMI cutoff points proposed for BMI classes. NW: Normal weight, IW: Ideal weight, OW: Overweight, MO: Mild obesity, SO: Severe obesity, NR: No risk of obesity, RO: Risk of obesity, O: Obesity, NA: Normal adiposity, IA: Ideal adiposity, AH: Adipose hypertrophy.

**Table A2:** Cutoff points proposal for FMI in adults.

FMI (kg/m^2^) Cutoff Points ∝ for Every BMI Class (kg/m^2^)				
*Sex*	Men		Women	
*Class of Weight*	BMI	FMI	BMI	FMI
Incompatible with life *	≤13.0	0	≤11.0	0
Thinness–Lipodystrophy	13.1–18.4	0.1–1.2	11.1–18.4	0.1–3.8
Normal weight–Normal adiposity	18.5–24.9	1.3–4.8	18.5–24.9	3.9–7.9
Ideal weight–Ideal adiposity	22.0	3.3	22.0	6.0
Overweight–Adipose hypertrophy	25.0–29.9	4.9–9.8	25.0–29.9	8.0–12.9
Obesity class I	30.0–34.9	9.9–14.8	30.0–34.9	13.0–17.9
Obesity class II	35.0–39.9	14.9–19.8	35.0–39.9	18.0–22.9
Obesity class III	≥40.0	≥19.9	≥40.0	≥23.0

Table A2. Ideal weight from Ramírez-López et al. (2018) [26]. General data for universal BMI cutoff points from WHO expert consultation (2004) [12]. * Henry (2001) [31].
